# Acetone Ingestion Mimics a Fasting State to Improve Glucose Tolerance in a Mouse Model of Gestational Hyperglycemia

**DOI:** 10.3390/ijms222312914

**Published:** 2021-11-29

**Authors:** Sandra Szlapinski, Brenda Strutt, Madeline Deane, Edith Arany, Jamie Bennett, David J. Hill

**Affiliations:** 1 Lawson Health Research Institute, St. Joseph Health Care, London, ON N6A 4V2, Canada; sandraszlapinski@hotmail.com (S.S.); Brenda.Strutt@lawsonresearch.com (B.S.); deanem2@mcmaster.ca (M.D.); edith.arany@lawsonresearch.com (E.A.); bennej6@mcmaster.ca (J.B.); 2 Department of Physiology and Pharmacology, Western University, London, ON N6A 3K7, Canada; 3 Molecular Biology and Genetics Program, Faculty of Science, McMaster University, Hamilton, ON L8S 4LD, Canada; 4 Departments of Pathology and Laboratory Medicine, Western University, London, ON N6A 3K7, Canada; 5 Department of Medicine, Western University, London, ON N6A 3K7, Canada; 6 Life Sciences Program, School of Interdisciplinary Science, McMaster University, Hamilton, ON L8S 4LD, Canada; 7 Departments of Paediatrics, Western University, London, ON N6A 3K7, Canada

**Keywords:** acetone, artemisinin, β-cell, islet of Langerhans, pancreas, pregnancy, glycemia

## Abstract

Gestational diabetes mellitus results, in part, from a sub-optimal β-cell mass (BCM) during pregnancy. Artemisinins were reported to increase BCM in models of diabetes by α- to β-cell conversion leading to enhanced glucose tolerance. We used a mouse model of gestational glucose intolerance to compare the effects of an artemisinin (artesunate) on glycemia of pregnant mice with vehicle treatment (acetone) or no treatment. Animals were treated daily from gestational days (GD) 0.5 to 6.5. An intraperitoneal glucose tolerance test was performed prior to euthanasia at GD18.5 or post-partum. Glucose tolerance was significantly improved in both pregnant and non-pregnant mice with both artesunate and vehicle-alone treatment, suggesting the outcome was primarily due to the acetone vehicle. In non-pregnant, acetone-treated animals, improved glucose tolerance was associated with a higher BCM and a significant increase in bihormonal insulin and glucagon-containing pancreatic islet cells, suggesting α- to β-cell conversion. BCM did not differ with treatment during pregnancy or post-partum. However, placental weight was higher in acetone-treated animals and was associated with an upregulation of apelinergic genes. Acetone-treated animals had reduced weight gain during treatment despite comparable food consumption to non-treated mice, suggesting transient effects on nutrient uptake. The mean duodenal and ileum villus height was reduced following exposure to acetone. We conclude that acetone treatment may mimic transient fasting, resulting in a subsequent improvement in glucose tolerance during pregnancy.

## 1. Introduction

Insulin resistance is a normal physiological feature in the third trimester of pregnancy that requires compensatory adaptations to occur within the maternal endocrine pancreas in order to maintain maternal euglycemia [1]. Should this compensation by the pancreatic β-cell be sub-optimal, gestational diabetes mellitus (GDM) can develop. GDM is diabetes that first appears during pregnancy and which normally regresses post-partum. Nonetheless, GDM is associated with adverse short-term outcomes to the offspring, such as macrosomia and respiratory distress syndrome, and longer-term issues such as childhood obesity and adult type 2 diabetes mellitus (T2DM) [2,3,4,5,6]. GDM is also a risk factor for subsequent T2DM in the mother. Current treatments for GDM, such as lifestyle behavioral change or administration of insulin or metformin, aim to decrease hyperglycemia but do not treat the underlying causes including a sub-optimal β-cell mass (BCM).

A reversible expansion of pancreatic BCM and α-cell mass (ACM) has been described in both rodents and humans, being maximal at late gestation (gestational day (GD) 18 in rodents) [7,8,9,10,11,12,13]. In rodents, the changes in BCM are known to be mediated by increased levels of placental lactogen and prolactin that initiate the proliferation of pre-existing β-cells [13,14]. Although evidence exists to support the expansion of BCM in pregnant humans, the mechanisms involved remain unclear [10,15]. New β-cells during pregnancy could potentially derive from the trans-differentiation of α-cells. Previous studies in non-pregnant animals reported that α-cells can replenish β-cells during β-cell stress by α- to β-cell transdifferentiation [16,17]. The use of structural analogs of artemisinins, a class of anti-malarial drugs, was reported to stimulate α- to β-cell conversion in vivo and in vitro [18] and improve glucose tolerance in non-pregnant animal models of diabetes [19,20]. The increased BCM resulted in improved glucose homeostasis, suggesting a possible beneficial effect of treatment with artemisinins in animal models of diabetes. Nonetheless, no data exist in hyperglycemic pregnancy. In this study, we investigated the effects of artemisinin treatment in a mouse model of gestational glucose intolerance during and following pregnancy [12]. Our hypothesis was that treatment with artesunate would improve glucose tolerance during pregnancy, as was observed in previous studies in diabetic mice [21]. However, we elucidated unexpected mechanisms responsible for improved glucose tolerance unrelated to the presence of an artemisinin.

## 2. Results

### 2.1. Artesunate Treatment in Early Gestation and Pregnancy Outcomes

**Figure 1:**
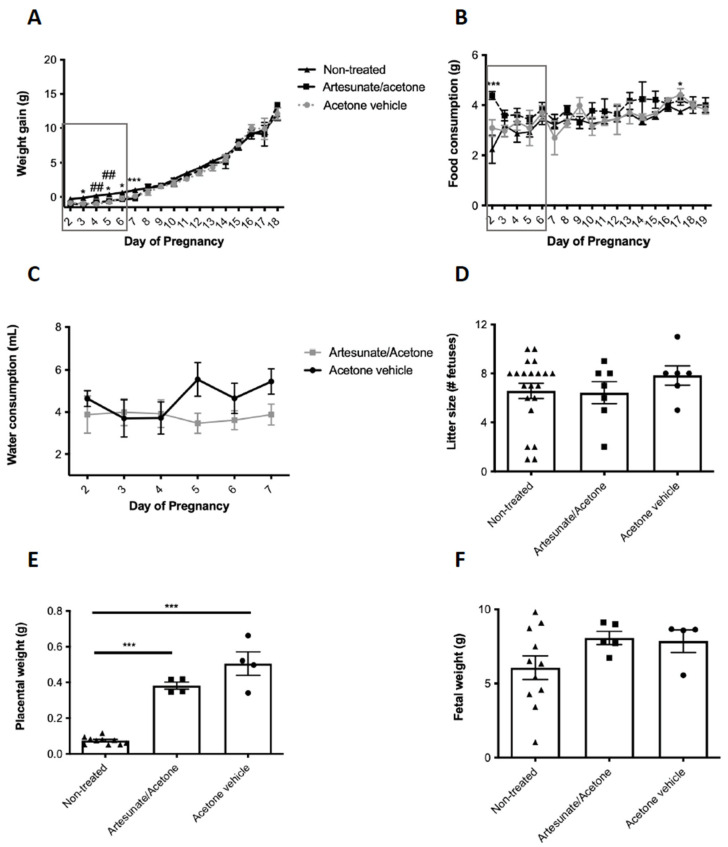
Weight gain during pregnancy (**A**), food consumption (**B**), water consumption (**C**), litter size **(D**), placental weight at GD18.5 (**E**), and fetal weight (**F**) in mice given artesunate/acetone (squares), acetone alone (circles) or no treatment (triangles). Values show mean ± sem. *n* = 4–6 animals per treatment group in (**A**–**C**) and *n* = 4–21 litters in (**D**–**F**). * *p* < 0.05, *** *p* < 0.001, non-treated vs. treatment group. ^##^ *p* < 0.01 non-treated vs. vehicle alone by an unpaired two-tailed Student’s *t* test (**A**–**C**) or by two-way ANOVA with a Bonferroni’s post-hoc test (**D**–**F**).

We examined pregnant mice previously exposed to a maternal low protein (LP) diet during pregnancy and lactation as a model of glucose intolerance during and following pregnancy [12]. Maternal weight gain was significantly lower in both artesunate/acetone and the acetone vehicle group alone compared to non-treated animals (Figure 1A). However, artesunate/acetone-treated animals had higher food consumption compared to non-treated animals, although this was observed only at GD2.5 and 17.5 (Figure 1B). Both treatment groups drank an average of 4mL of solution a day during treatment (Figure 1C), which is comparable to values for non-treated mice of between 3 and 4 mL depending on body weight [22]. There was no difference in the number of fetuses at GD18.5 (Figure 1D). However, placental weight was significantly higher in both artesunate/acetone and acetone vehicle animals compared to non-treated animals (Figure 1E). Furthermore, there were no significant differences in fetal weight between treatment groups at GD18.5 despite there being a non-significant trend suggesting animals in the artesunate/acetone-treated group weighed more than non-treated animals (*p* = 0.06) (Figure 1F).

### 2.2. Both Artesunate- and Acetone Vehicle-Treated Animals Have Improved Glucose Tolerance vs. Non-Treated Females

**Figure 2:**
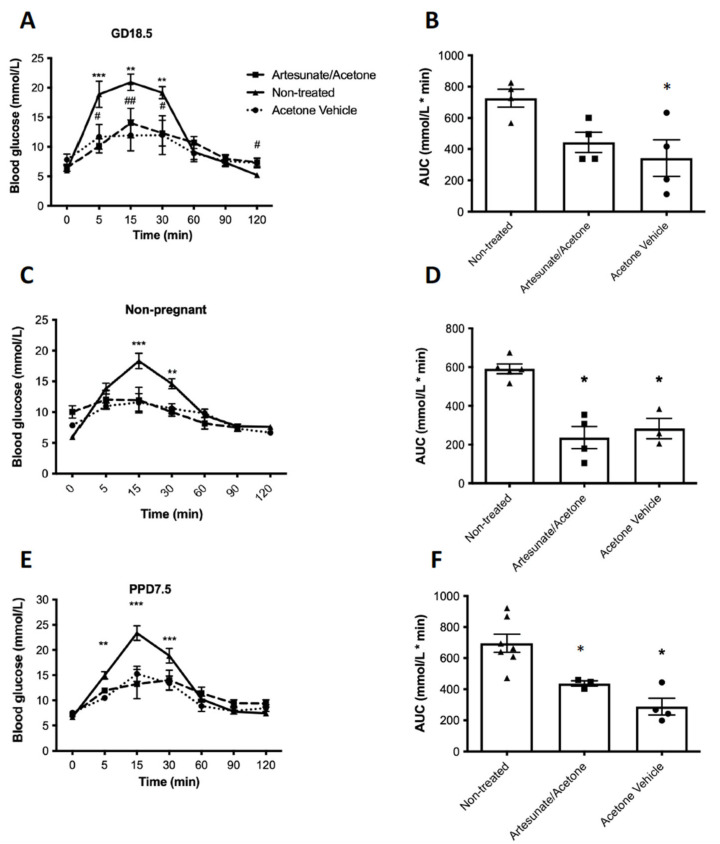
Blood glucose values during a glucose tolerance test and the resulting area under the curve (AUC) for pregnant (GD 18.5, (**A**,**B**)), non-pregnant (**C**,**D**), or post-partum (**E**,**F**) mice previously treated with artesunate/acetone (square), acetone vehicle alone (circle), or no treatment (triangle). Values show mean ± sem. *n* = 4 animals at GD18.5 per treatment group and 4-5 in non-pregnant and PPD7.5 animals. * *p* < 0.05, ** *p <* 0.01, *** *p* < 0.001 non-treated vs. treatment group. ^#^ *p* < 0.05, ^##^ *p* < 0.01 non-treated vs. vehicle by a one-way ANOVA with Tukey’s post-hoc test.

Fasted blood glucose (4 h fast) did not significantly differ with treatments for non-pregnant, pregnant, or post-partum mice. Pregnant mice in both the artesunate/acetone and acetone vehicle group had significantly lower blood glucose levels at 5, 15, and 30 min during the IPGTT relative to non-treated animals at GD18.5 (Figure 2A). At 120 min, the acetone vehicle-treated animals had significantly higher blood glucose levels compared to non-treated animals. Furthermore, the area under the glucose tolerance curve (AUC) was significantly lower in the artesunate/acetone and acetone vehicle groups (Figure 2B). IPGTT glycemic excursions were also significantly lower following artesunate/acetone or acetone vehicle treatment compared with non-treated mice in non-pregnant and PPD7.5 animals (Figure 2C,E), as were the AUC values (Figure 2D,F), leading to the conclusion that the acetone vehicle was primarily responsible for improved glucose tolerance.

### 2.3. Acetone Treatment Alters Pancreas Histology during and after Pregnancy

**Figure 3:**
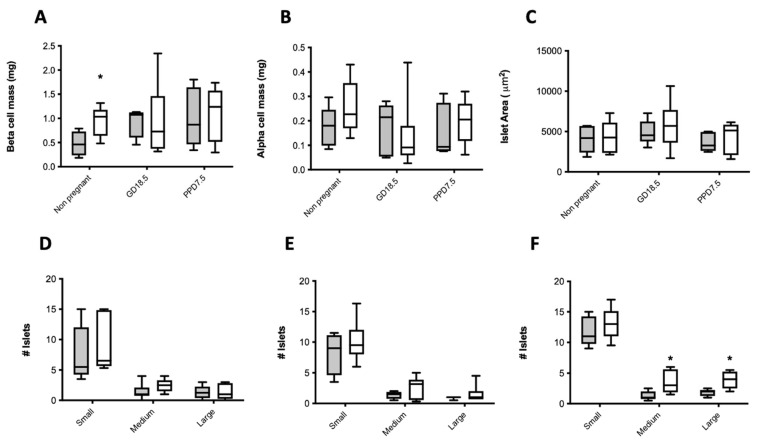
BCM (**A**), ACM (**B**), and mean islet area (**C**) in non-pregnant, pregnant (GD18.5), or post-partum mice previously treated with water alone (shaded bars) or acetone (open bars). The distribution of islet sizes (small—<5000 μm^2^; medium—5000–10,000 μm^2^; or large—>10,000 μm^2^) is shown below for each of the above groups of mice (**D**–**F**). Medium values are shown, *n* = 4–8 animals per treatment group. * *p* < 0.05, acetone-treated vs. water by two-way ANOVA with a Bonferroni’s post-hoc test.

Beta-cell mass did not differ between artesunate and vehicle-treated mice, and data were subsequently combined for analysis. Beta-cell mass was significantly higher in non-pregnant acetone-treated animals compared to non-treated mice (Figure 3A). There were no significant differences in ACM (Figure 3B) or mean islet size (Figure 3C) between treatment groups over time. Furthermore, no differences in mean islet size distribution were found between treatment groups in non-pregnant (Figure 3D) or pregnant GD18.5 (Figure 3E) animals. However, acetone-treated animals at PPD7.5 had significantly more medium and large-sized islets compared to non-treated mice (Figure 3F).

### 2.4. Acetone Treatment Causes Hyperglucagonemia during and after Pregnancy

**Figure 4:**
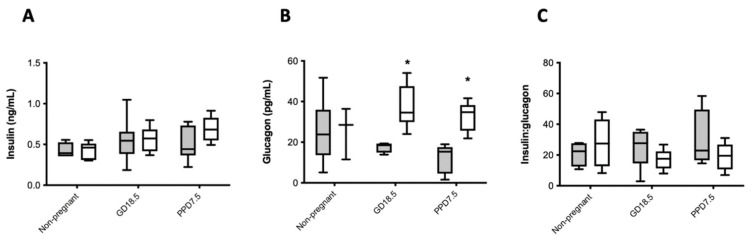
Serum insulin (**A**), glucagon (**B**), and serum insulin to glucagon ratio (**C**) in non-pregnant, pregnant (GD18.5), or post-partum mice previously treated with water alone (shaded bars) or acetone (open bars). Medium values are shown, n = 3–8 animals per treatment group. * *p* < 0.05, acetone-treated vs. water by two-way ANOVA with a Bonferroni’s post-hoc test.

Serum insulin and glucagon were quantified from blood collected *via* cardiac puncture at the end of the IPGTT (120 min). Serum insulin and glucagon values did not differ between artesunate and vehicle-treated mice, and data were combined for analysis in this and subsequent studies. There were no significant differences in serum insulin between treatment groups over time (Figure 4A). However, serum glucagon levels were significantly higher at GD18.5 and PPD7.5 in acetone-treated animals compared to non-treated animals (Figure 4B). There were no significant differences in the serum insulin to glucagon ratio between treatment groups over time (Figure 4C).

### 2.5. Acetone Treatment Increases Islet Bihormonal Cell Number

**Figure 5:**
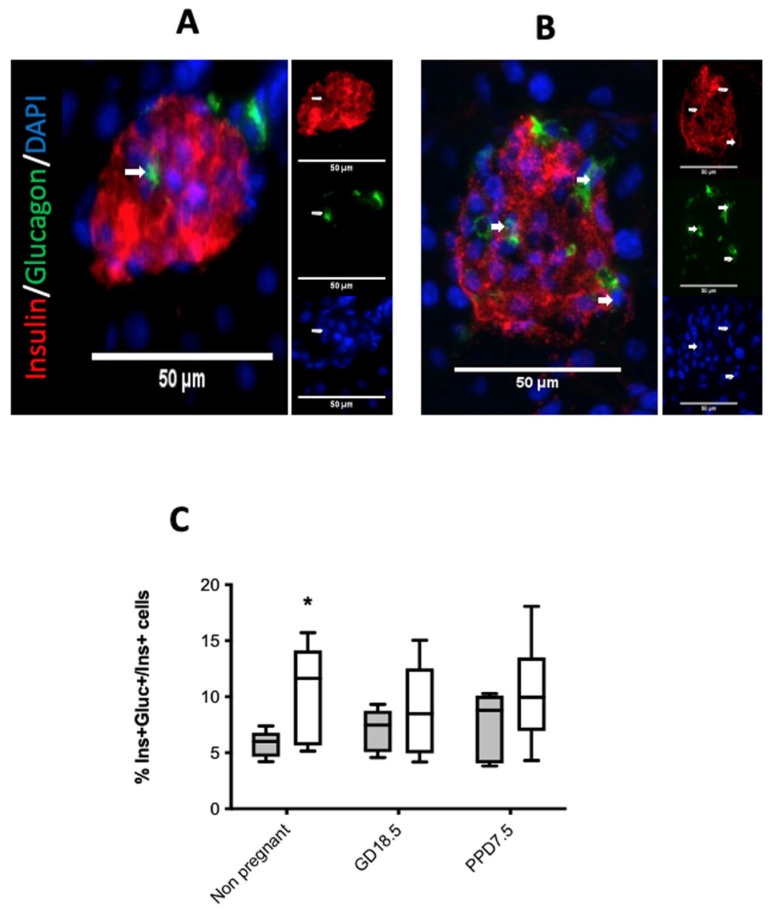
Representative micrographs of insulin and glucagon dual staining islet cells in a non-pregnant mouse treated with water alone (**A**) or with acetone (**B**). The percentage of dual staining cells relative to all insulin-staining cells is shown in (**C**) for non-pregnant, pregnant (GD18.5), or post-partum mice previously treated with water alone (shaded bars) or acetone (open bars). Values show medians, *n* = 5–8 animals per treatment group. * *p* < 0.05, acetone-treated vs. water by two-way ANOVA with a Bonferroni’s post-hoc test.

To investigate a potential mechanism underpinning the new β-cells observed in non-pregnant acetone-treated animals, we quantified insulin and glucagon double-positive cells (Figure 5A,B) as a marker for possible α- to β-cell trans-differentiation. Acetone-treated non-pregnant animals had significantly more bihormonal cells compared to non-treated animals (Figure 5C), but this was not the case for pregnant or post-partum mice.

### 2.6. Acetone Treatment Increases Placental Apelinergic Gene Expression

**Figure 6:**
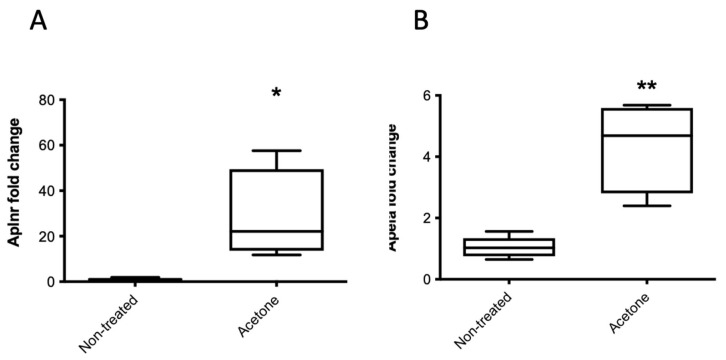
Placental mRNA expression of Aplnr (**A**), and Apela (**B**) in acetone-treated or non-treated pregnant mice (GD18.5). The fold change in expression was measured relative to the housekeeping gene cyclophilin A. Median values are shown, *n* = 5 non-treated animals and 4 acetone-treated animals. * *p* < 0.05, ** *p* < 0.01, acetone-treated vs. water by a one-way ANOVA with Tukey’s post-hoc test.

To investigate a potential mechanism of improved glucose tolerance in pregnant acetone-treated animals, we analyzed the placenta, since placental weight was higher in acetone-treated animals (Figure 1E). Considering the placenta secretes apelin and apela which are known to alter β-cell number we examined the expression of the apelinergic system. Relative Aplnr (Figure 6A) and Apela (Figure 6B) mRNA levels were both significantly higher in acetone-treated animals compared to non-treated mice.

### 2.7. Acetone Treatment Alters Gut Morphology

**Figure 7:**
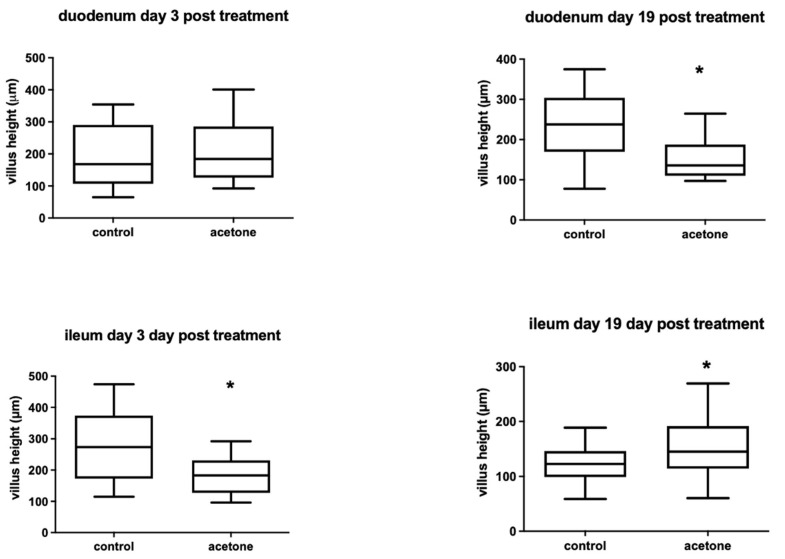
Median villus height in transverse tissue sections of duodenum or ileum at 3 or 19 days following administration of acetone in drinking water, or water alone. *n* = 19–76 separate villi for each time point and condition, each measured from 3 animals. * *p* < 0.001, acetone-treated vs. water by a one-way ANOVA with Tukey’s post-hoc test.

The improved glucose tolerance in acetone-treated animals could have reflected a transient or longer-term effect on gastrointestinal function, resulting in impaired nutrient uptake equivalent to a period of fasting. This was investigated by examining tissue morphology in two areas of the gastro-intestinal tract, the duodenum and the ileum, in adult female mice either 3 or 19 days after administration of acetone within drinking water. Gross morphology of the villi did not differ following acetone treatment at either time point, nor did the number of villi per transverse tissue section. However, the mean villus height was significantly reduced 3 days after acetone treatment in the ileum but not in the duodenum (Figure 7). Conversely, 19 days after treatment, the villus height had fully recovered and was greater than in controls in the ileum but was significantly reduced in the duodenum. Therefore, acetone treatment had both short- and long-term effects on villus morphology but in different regions of the gut.

## 3. Discussion

Artemisinins were shown to increase BCM *via* α- to β-cell trans-differentiation and improve glucose homeostasis in non-pregnant animal models of diabetes [18], although these findings are controversial and have been rebutted [21,23]. Our experiments showed a high consumption of artesunate within drinking water and no indications of fetal resorptions to implicate embryolethality. Nonetheless, the initial objective of testing artesunate was negated as we noted that the improvement in glucose tolerance was similar between the treatment group of artesunate diluted in acetone vehicle and that of the vehicle alone, leading us to conclude that the effect was primarily due to administration of the acetone vehicle. Thus, we re-adjusted our focus to determine the effects of acetone on glucose homeostasis and pancreas histology.

Weight gain was lower in acetone-treated animals during treatment, although body weight recovered by the end of the experiment. Acetone-treated animals consumed a comparable amount of food as non-treated animals, presenting the possibility that acetone might have had a transient detrimental effect on nutrient uptake *via* the intestines, resulting in reduced weight gain in treated animals. A previous study found that acetone abolished the adhesion of F18-fimbriated (F18R) *E. coli* to isolated porcine intestinal villi in vitro, concluding that F18R was a glycolipid [24]. Since glycosphingolipids (GSL) are a major component of intestinal enterocytes, it is possible that acetone could be breaking down the intestinal villi and limiting nutrient absorption. In an animal model with genetic deletion of the gene for the enzyme that catalyzes the initial step of GSL biosynthesis (*Ugcg*), newborn mice presented with growth retardation and loss of body fat deposits due to a severe disturbance in the uptake of nutrients [25]. The same study showed that adult mice had a drastic decrease in body weight, as was observed in our study following treatment with acetone. It was concluded that GSLs in the intestinal epithelium are essential for intestinal endocytic function to effectively absorb nutrients. It is worth noting that reduced nutrient and glucose uptake for the time period of the treatment in our study could mimic a situation of fasting, which has been suggested to have protective effects by reducing oxidative stress, and protects against many diseases in both rodents and humans [26,27]. While the gross anatomy of the villi in the duodenum and ileum in the present study was not altered after acetone ingestion, there were both short- and long-term effects on villus height, suggesting a disruption to endocytic generative cycles and, potentially, function.

It is also possible that acetone crossed the gastrointestinal epithelium and altered metabolic homeostasis at peripheral tissues. Orally administered acetone is very rapidly absorbed into the blood stream in both rodents and humans. After administration of ^14^C-labelled acetone to rats by gavage once a day for 7 days, between 67 and 76% was expired as CO_2_ and 7% as acetone in the urine over 24 h, indicating an absorption efficiency of around 80% [28]. Once within the blood, acetone is equally distributed within the tissues and no immediate endocrine effects have been reported. It is possible to estimate the likely steady state plasma levels of acetone during oral administration based on previous reports. Scholl and Iba [29] administered 7.5% *v*/*v* acetone within drinking water for 11 days. After day 4, a plateau of plasma acetone was achieved around 1200 μg/mL. Based on the 0.4% *v*/*v* acetone dilution used in the present study, this would equate to a plasma concentration of around 64 μg/mL, as there is a linear correlation between acetone dosage and the amount absorbed. However, since acetone is readily soluble in water, its absorption through the gastrointestinal tract is compatible with impaired active epithelial transport of glucose and other nutrients through enterocytic damage.

Further investigations of mechanisms of improved glucose tolerance in acetone-treated animals examined changes in the endocrine pancreas. Although BCM was higher in non-pregnant acetone-treated animals compared to controls, this did not correlate with higher serum insulin levels. Serum glucagon levels were higher at the end of the IPGTT at both GD18.5 and PPD7.5, despite no differences being observed in ACM. Adult mice with *Ugcg* gene deletion displayed severe structural defects in the small and large intestine, which could also extend to the enteroendocrine cells [25]. The L-cells in the distal ileum and colon secrete glucagon-like peptide 1 (GLP-1), an incretin hormone that is released in response to nutrient ingestion. GLP-1 increases insulin secretion and inhibits glucagon secretion [30]. The elevated levels of serum glucagon at GD18.5 and PPD7.5 could suggest damage to the enteroendocrine cells in acetone-treated animals, thereby limiting the release of GLP-1.

As an alternate hypothesis, we quantified bihormonal (insulin and glucagon double-positive) cells within islets as a marker for possible α- to β-cell trans-differentiation that could contribute to an increase in BCM. There was an increased percentage of bihormonal cells in non-pregnant acetone-treated animals compared with controls. These findings are in agreement with those observed following transient fasting of non-pregnant mice, where a greater number of transitional α- to β-cells were observed upon re-feeding [31], resulting in β-cell regeneration and a rescue from diabetes. However, we previously found that α- to β-cell trans-differentiation did not contribute to the increased BCM that normally occurs during mouse pregnancy [32]. It is possible that the bihormonal cells represent an intermediate stage in the functional maturation of resident β-cell progenitors, which we previously showed to contribute to the increased BCM of pregnancy in mice [11]. Determination as to whether an increased contribution of β-cell progenitors to the improved glycemic control following acetone exposure will require analysis much earlier in gestation.

Placentae were also analyzed as contributing to improved glucose tolerance since placental weight was higher in acetone-treated compared to non-treated animals. A previous study showed increased deposition of glycogen in placentae from women with GDM, as the placenta acts as a buffer for excess glucose, thereby lowering blood glucose levels in the mother [33]. Interestingly, the apelinergic system was shown to promote the transplacental transport of glucose from mother to fetus in rat dams injected intravenously with apelin-13 without changes to the expression of the placental glucose transporters Glut1 and Glut3 [34]. Rather, it was reported that at mid to late gestation, apelinergic signaling increased the vasodilation of fetal arterioles and glucose transport to the fetus. In the present study, expression levels of apelinergic genes (Apela and AplnR) were increased in the large placentae of acetone-treated animals at GD18.5 compared to non-treated animals. Therefore, the transfer of glucose from mother to fetus in our study could be enhanced by upregulation of the placental apelinergic system, resulting in an improved glucose status in the mother. The trend towards higher fetal weight in acetone-treated animals further supports this possibility of greater transplacental glucose transfer. Apelin has also been linked to placental growth and efficiency since fetal apelin levels were reduced following maternal food restriction [35]. Furthermore, we recently reported a decreased presence of the Aplnr in the endocrine pancreas in hyperglycemic mouse pregnancies, associated with a reduced BCM, using the same model as utilized in the present study [36].

**Figure 8:**
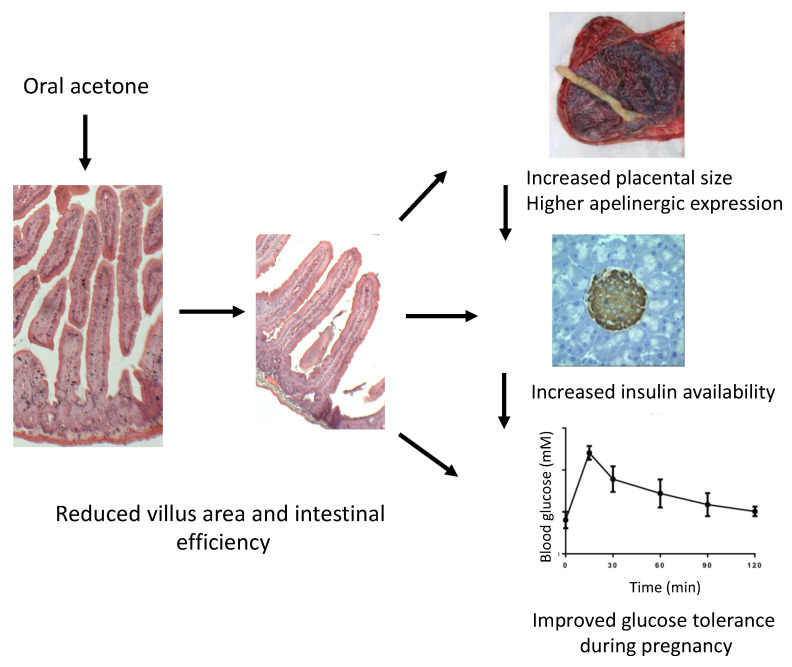
Possible mechanisms whereby transient acetone ingestion during pregnancy might improve maternal glucose tolerance. Acetone ingestion resulted in long-standing changes in villus length in the intestinal tract. This was associated, during pregnancy, with increased placental weight and increased expression of the placental apelinergic axis. Apelinergic ligands may potentiate pancreatic endocrine cell remodeling to increase insulin availability, resulting in improved glucose tolerance.

In conclusion, acetone treatment improved glucose tolerance in non-pregnant, pregnant, and post-partum mice. In non-pregnant animals, improvements in glucose tolerance were associated with an increased BCM, possibly involving α- to β-cell conversion. However, in pregnant animals, the improved glucose tolerance could involve compensatory mechanisms such as an upregulation of the placental apelinergic system, resulting in vasodilation and increased glucose transfer subsequently decreasing maternal blood glucose levels. The possible interactions of these mechanisms resulting in improved glucose tolerance are shown in Figure 8. Our findings provide a potential therapeutic glucose-lowering effect of acetone *via* the mimicry of short-term fasting to improve glucose tolerance, including in a model of gestational glucose intolerance. The use of oral acetone in a human health context is not practical, especially during pregnancy, given the association with gut epithelial damage. However, other ketones such as β-hydroxybutyrate may be worthy of future study. In a human context, these results suggest that nutritional moderation very early in pregnancy in women at risk of gestational diabetes may improve glucose tolerance in third trimester, possibly due to an upregulation of the apelingeric system in placenta, resulting in greater maternal insulin availability and increased vasodilation of fetal arterioles and glucose transport to the fetus, thereby lowering maternal blood glucose levels [34].

## 4. Materials and Methods

### 4.1. Animals, Treatment, and Sample Collection

All animal procedures were approved by the Animal Care Committee of Western University in accordance with the guidelines of the Canadian Council for Animal Care. The studies were compliant with the ARRIVE guidelines both in the design and reporting of the findings. Mice were housed in a temperature-controlled room with a 12-h light:dark cycle at Lawson Health Research Institute, London, ON, Canada. Water and food were given *ad libitum.* A total of 35 adult C57BL/6 male and female (F0) (6-week-old) mice were obtained from Charles River Laboratories (Wilmington, MA, USA).

**Figure 9:**
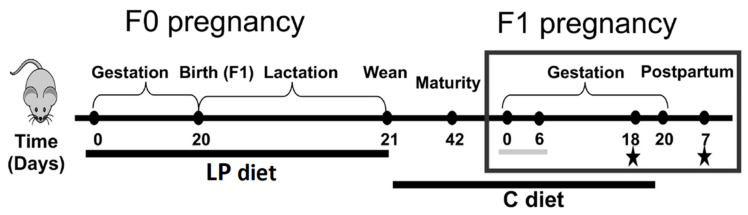
Mouse model of glucose intolerance in pregnancy. F0 dams were fed a low protein (LP) diet (8% protein) during gestation and lactation. Offspring (F1) were weaned onto a control diet (C, 20% protein). At maturity, F1 female offspring were time-mated with C-fed males. Artesunate (1 mg/mL) or acetone vehicle groups of pregnant females were treated from gestational day (GD) 0.5 to 6.5 (grey bar) with drug or vehicle administered through the drinking water. Non-treated animals were given regular tap water. Non-pregnant animals were age-matched to females in the pregnant group. Stars indicate timepoints where an intraperitoneal glucose tolerance test was performed prior to euthanasia, and the pancreas and/or intestine was removed for fluorescence immunohistochemistry.

Mice showing glucose intolerance at GD18.5 and post-partum were generated using a previously described protocol involving a low protein dietary insult during early life [12]. Briefly, F0 females underwent estrous cycling and were time-mated with males. Dams were fed a low protein (LP, 8% protein, Bio-Serv, Frenchtown, NJ, USA) diet similar to that described by Snoeck et al. [37] throughout gestation and lactation. Female offspring (F1) were weaned onto a control diet (C, 20% protein) for the remainder of the study. At maturity (postnatal day, PND, 42), female offspring (F1) of LP diet-fed mothers were randomly allocated into two study groups: pregnant (GD18.5 or postpartum day (PPD) 7.5) or non-pregnant. We chose GD18.5 based on previous findings that this was a time point where glucose intolerance and reduced BCM were present. We also investigated mice after parturition at PPD7.5 due to findings from a previous study showing that glucose intolerance persisted until 1 month post-partum in this model [6]. These animals were subsequently separated into an artemisinin-treated group, vehicle group, or non-treated group (non-treated non-pregnant and GD18.5 data retrieved from [12], non-treated PPD7.5 data retrieved from [6]). All pregnant-grouped females were time-mated with control diet-fed males. We adapted a protocol from a previous study where mice were treated with artesunate in an acetone vehicle diluted in drinking water daily, which resulted in an increased islet size [18]. A stock solution of 250 mg/mL artesunate (Cayman Chemicals, Ann Arbor, MI, USA) in acetone (Sigma-Aldrich, St. Louis, MO, USA) was prepared daily, 40 μL of which was diluted daily in 10 mL drinking water for a final concentration of 1mg/mL artesunate. An equal concentration of acetone was used in the control group, and drinking water was provided *ad libitum*. Water bottles were covered with aluminum foil to prevent light penetration [18]. Vehicle or treatment were replaced daily from GD 0.5 to 6.5, after which the solution was replaced with tap water for the remainder of the experiment (Figure 9). Females were euthanized by CO_2_ asphyxia at their assigned day for comparison to non-pregnant age-matched females. Maternal pancreatic samples were fixed in 4% paraformaldehyde for histology and embedded in optical cutting temperature compound. Maternal serum samples were collected *via* cardiac puncture. Placenta samples were collected in 1mL of RNAlater RNA Stabilization Reagent and frozen at −20 °C (Qiagen, Hilden, Germany). Duodenum and ileum were also removed from non-pregnant adult female mice either 3 or 19 days after administration of acetone or water alone, tissues were fixed in 10% formalin and embedded in paraffin, and transverse sections were prepared for histology.

### 4.2. Intra-Peritoneal Glucose Tolerance Test

Prior to euthanasia, an intra-peritoneal glucose tolerance test (2 g glucose/kg body weight, IPGTT) was performed on all animals. Mice were fasted for 4 h. Blood glucose was measured from the tail at 0, 5, 15, 30, 60, 90, and 120 min using a One Touch Ultra2 glucometer.

### 4.3. Immunohistochemistry and Tissue Morphometry

Fixed pancreas tissue was prepared and sectioned as previously described [38]. At least two 7 μm-thick replicate cryosections were cut from each pancreas with an interval between each section >100 μm representing at least two longitudinal slices through the pancreas. Sections included both the head and tail of the pancreas. Immunofluorescence immunohistochemistry was performed to localize insulin and glucagon as described previously [12]. Antibodies against insulin (1:200, anti-mouse, Sigma-Aldrich) and glucagon (1:200, anti-rabbit, Santa Cruz Biotechnology, Dallas, TX, USA) were applied to tissues and incubated overnight at 4 °C. The following day, secondary antibodies (1:500 Thermo Fisher Scientific Waltham, MA, USA) were applied against the primary antibody using 555 and 488 fluorophores, respectively, along with DAPI (1:500, Thermo Fisher Scientific) to counterstain nuclei.

Tissue sections were visualized at 20x using a Nikon Eclipse TS2R inverted microscope (Nikon, Minato, Tokyo, Japan) with the NIS-Elements program (Nikon, Minato, Tokyo, Japan), and images were captured and analyzed using the Cell Counter plugin in ImageJ software. Every insulin- and glucagon-expressing cell was imaged for each section and for each animal. Manual cell counting analysis determined the percentage of bihormonal, Insulin^+^ Glucagon^+^ cells as a marker for α- to β-cell transitional cells [13,39,40,41]. To determine BCM and ACM, morphometric analysis was performed by manually measuring the total pancreas area for each tissue section, and the relative area of β-cells and α-cells [12,42]. BCM and ACM were calculated by multiplying the total β or α-cell volume (sum of entire β or α-cell area/surface area of the entire tissue section) by the pancreas weight. Islets were counted per tissue section and further separated by size into small (<5000 μm^2^), medium (5000–10,000 μm^2^), or large (>10,000 μm^2^) islets as previously described [12,35].

Sections of duodenum or ileum were stained for hematoxylin and eosin (Hematoxylin and Eosin Stain Kit, Vector Laboratories (cat# H-3502), Burlingame, CA, USA). The height of the villus from the base of the crypt to the tip of the villus was recorded for all villi present in a tissue section and from two separate sections from each animal. Only complete villi present within the transverse plane of the tissue were considered.

### 4.4. Serum ELISA Assays

Maternal (F1) blood serum was used to quantify insulin and glucagon using an Ultra-Sensitive Mouse Insulin ELISA kit (Crystal Chem, Downers Grove, IL, USA) and Mouse Glucagon ELISA kit (Crystal Chem), respectively. The insulin assay has a sensitivity of 0.05 ng/mL using a 5µL sample with precision CV ≤ 10.0%. The glucagon assay has a sensitivity of 1.1 pg/mL using a 10uL sample with precision CV < 10%. Data were collected using a BioRad iMark plate reader and analyzed using Microplate Manager Software.

### 4.5. Quantitative Polymerase Chain Reaction

Placenta samples (3-5mg) were minced with scissors in lysis buffer and Qiashredder spin columns (Qiagen) prior to total RNA extraction using the RNeasy Plus Micro kit (Qiagen). Total RNA (<1 μg) was extracted and reverse transcribed using iScript Reverse Transcription Supermix (Bio-Rad Laboratories, Hercules, CA, USA). Quantitative PCR experiments were accomplished using the 2^−ΔΔC^_T_ method after confirmation of parallel PCR amplification efficiencies between each gene of interest and the housekeeping gene Cyclophilin A (Mm02342429_g1, Applied Biosystems, Forest City, CA, USA). The apelin receptor and apela mRNA levels were quantified using the TaqMan gene expression assay and the TaqMan Fast Advanced Master Mix (Invitrogen, Carlsbad, CA, USA) with the following Taqman primers: apelin receptor (Aplnr) (Mm00442191_s1, Applied Biosystems) and Apela (Mm04278372_m1, Applied Biosystems). PCR reactions were run in triplicate using the Quantstudio design and analysis software. Quantstudio 5 (Applied Biosystems) was programmed with the following thermal-cycling profile: polymerase activation step at 95 °C for 20 s, followed by denaturation at 95 °C for 3 s, and annealing/extension at 60 °C for 30 s for 40 cycles. Levels of mRNA expression were calculated relative to those of the housekeeping gene cyclophilin A.

### 4.6. Statistical Analysis

Normality of distribution for each data set was determined by the Shapiro–Wilk test with a QQ plot. Normally distributed data are presented either as mean ± SEM, or as box plots showing median values with the first and third quartiles representing the minimum and maximum values. Statistical significance was analyzed using GraphPad Prism software (Version 5.0). An unpaired two-tailed Student’s *t* test, one-way ANOVA, or two-way ANOVA were applied according to the set of groups that were compared. Tukey’s post-hoc test or a Bonferroni’s post-hoc test was performed after one-way ANOVA or two-way ANOVA analysis, respectively. Each animal constituted a single unit of analysis (*n*). *n* = 4–8 animals per treatment per timepoint. Statistical significance was determined as *p* < 0.05.

## Data Availability

The datasets generated and analyzed during the current study are available from the corresponding author on reasonable request.
